# Acute Promyelocytic Leukemia (APL): Comparison Between Children and Adults

**DOI:** 10.4084/MJHID.2014.032

**Published:** 2014-04-15

**Authors:** Anna Maria Testi, Mariella D’Angiò, Franco Locatelli, Andrea Pession, Francesco Lo Coco

**Affiliations:** 1 Department of Cellular Biotechnologies and Hematology, Sapienza University of Rome, Italy; 2 Department of Pediatric Hemato-Oncology, IRCCS Ospedale Bambino Gesù, Roma University of Pavia, Italy; 3 Department of Pediatric Hemato-Oncology, University of Bologna, Italy; 4 Department of Biomedicine and Prevention, University Tor Vergata, Rome, Italy; 5 Laboratory of Neuro-Oncoematology, Santa Lucia Foundation, Rome, Italy

## Abstract

The outcome of adults and children with Acute Promyelocytic Leukemia (APL) has dramatically changed since the introduction of all trans retinoic acid (ATRA) therapy. Based on the results of several multicenter trials, the current recommendations for the treatment of patients with APL include ATRA and anthracycline-based chemotherapy for the remission induction and consolidation, and ATRA combined with low-dose chemotherapy for maintenance. This has improved the prognosis of APL by increasing the complete remission (CR) rate, actually > 90%, decreasing the induction deaths and by reducing the relapse rate, leading to cure rates nowadays exceeding 80% considering both adults and children.^1^–^9^ More recently the combination of ATRA and arsenic trioxide (ATO) as induction and consolidation therapy has been shown to be at least not inferior and possibly superior to ATRA plus chemotherapy in adult patients with APL conventionally defined as non-high risk (Sanz score).^10^

Childhood APL has customarily been treated on adult protocols. Data from several trials have shown that the overall outcome in pediatric APL appears similar to that reported for the adult population; however, some clinical and therapeutic aspects differ in the two cohorts which require some important considerations and treatment adjustments.

## Epidemiology of Pediatric APL Compared to that of Adults

In childhood, APL is very rare disease; its incidence seems to be different according to various geographic areas. In the United States, as in Central and Northern Europe, the percentage of APL patients is 5–7% of all pediatric acute myeloid leukemia (AML) cases; a higher frequency (about 20%) is reported in children of Latino/Hispanic descent.^6^–^8^;^11^–^16^ In the AML Berlin-Frankfurt-Münster (BFM) studies, 8–10 APL children were registered per year, compared to 16–18 pediatric patients treated, each year, within the Gruppo Italiano Malattie Ematologiche dell’Adulto (GIMEMA)-Associazione Italiana Ematologia Oncologia Pediatrica (AIEOP) AIDA trials.^6^,^14^,^17^ Both in the Latino/Hispanic and in Western countries, however, the number of adult APL is much higher compared to that of children, like it is observed in the other forms of AML (110–120 new adult APL cases/a year, in Italy).^5^,^18^ Consequently, pediatric patients have represented only a minimum percentage in trials enrolling both children and adults. Between November 1996 and June 2004, the Programa Espanol de Tratamientos en Hematologia (PETHEMA) LPA96 and LPA99 studies included 639 consecutive patients with newly diagnosed APL from Spain, Netherlands, Belgium, Argentina and the Czech Republic; 67 of them (10.5%) were aged less than 18 years.^2^,^3^ In the Italian GIMEMA AIDA-0493 and -2000 protocols, a total of 1095 adults with APL (age 18–61) were included; in this time frame, a total of 247 (22.6%) children received the same treatments.^5^,^6^,^17^,^18^

Data from single institutions, as well as population-based study, suggested that the Latino-American population has a higher proportion of APL among AML diagnoses, which account for as much as 37.5%. In centers of Brazil, it has been reported that APL represents 28.2% of all AML cases, a fraction that is very similar to that reported by Melo et al (28%) in another Brazilian center. These figures have been confirmed by information from Mexico (20%), Venezuela (27.8%) and Peru (22%). The increased incidence has also been reported in pediatric age.^19^,^22^

In China, the lack of population-based registries makes it difficult to determine the real incidence of APL, which is estimated on the basis of its relative frequency among other AML subtypes in large clinical trials. According to data so far published, it appears that the Chinese population has a higher prevalence of APL when compared to most non-Chinese studies 23,24. The most striking ethnic difference is evident in children. In a single-center large series of 629 Chinese patients with de novo AML, 138 (22%) were diagnosed as having APL, cytogenetically confirmed; the incidence of APL was higher in the pediatric age (34%) compared to both adults (19%) and elderly (3%). In Peking Union Medical College, 51 (31.6%) of the 141 cases of pediatric AML, registered between 1996 and 2004, were diagnosed as having APL; in the Children’s hospital of Zhejiang University School of Medicine, between 1997 and 2005, 49 (26.5%) of the 185 newly diagnosed pediatric AML, were APL. The percentage of APL could be higher in children than in adults, in this country, but a population-based cancer registry would be necessary to confirm this data.^23^–^25^ Also in low-income countries such as Iraq, the real incidence of pediatric (< 15 years) APL is still unknown; it is estimated on the basis of its relative frequency among other pediatric AML subtypes diagnosed at a single institution. At the Pediatric Oncology Unit, College of Medicine, in Baghdad, which represents a referral center for childhood cancer in Iraq, the overall high incidence of childhood APL is recorded: approximately 30% of pediatric AML are morphologically diagnosed as APL.^26^ The same high incidence is not registered among adult and elderly patients (age > 15 years), but in this country many adult patients with leukemia are not referred to a specific oncology center. Thus, the presumed higher prevalence of APL may reflect underestimation of AML cases. Indeed, it is difficult to identify the reasons behind the apparent high prevalence of Iraqi childhood APL; in fact, epidemiological and environmental studies are not carried out, and pediatric and adult cancer registries are still not available.

Ethnic variability may also account for the different incidence of APL in the various countries; environmental factors may play a role; however, the incidence in the different age groups is still not explained. In addition, the diagnostic poor facilities, have to be considered as possible bias for the reported higher incidence of childhood APL in the developing countries.

## Are there Clinical and Biological Differences in Pediatric APL Compared to Adults?

Some pediatric trials have shown clinical and biological differences between adults and pediatric APL. Pediatric APL is diagnosed at a median age of 9–12 years; diagnosis at age < 1 year is very rare in all countries. In our GIMEMA-AIEOP AIDA-0493 study, one of the 124 children was under 2 years, and only one of the successive 123 children treated with GIMEMA AIDA-2000 protocol was aged < 12 months.^6^,^17^ Among the 66 children included in the PETHEMA LPA96 and LPA99 studies, only 6 of them were less than 3 years old (9%).^7^ The BFM-AML Study Group reported 81 children and adolescents treated with three consecutive protocols (AML-BFM-93, -98 and -2004 studies); only one of them was under 1 year of age and 4% (2 cases) of the 53 children of the first North American Intergroup trial (INT0129) were aged ≤ 2 years.^11^,^14^ APL seems to be more frequent in children with an age ≥ 10 years: 61% in the PETHEMA studies, 50% in the Italian GIMEMA-AIEOP AIDA and 65% in the BFM protocols.^6^,^7^,^14^ A higher frequency of APL in older children has been also described in two small series of Chinese patients (10/19 and 18/37 children aged over 10 years, in the two studies, respectively).^23^,^24^ However it should be noted that, in the pediatric APL series reported to date in the literature, the median age ranged widely, from 7.2 years in the German Austrian Swiss study to 15 years in the European APL study.^28^,^29^ In most countries, the incidence of the disease increases during the second decade of life reaching a plateau during early adulthood when the incidence remains constant, until it decreases after 60 years of age.

The female sex seems to be predominant among children but not in adults; the predominance of girls (71%) was reported in the French pediatric study published by de Botton et al., by the Children Cancer Group (CCG) and Pediatric Oncology Group (POG) (60%) and by the PETHEMA group (59%) but not in our GIMEMA-AIEOP large group that reported a female/male ratio of almost 1.^6^,^8^,^13^ An equally even distribution of the two sexes in children and adults had been previously noted by Guglielmi et al, in a series of 196 Italian APL patients.^30^

High body mass index (BMI) is more frequent in APL than in other AML subtypes, both in adults and children. Estey reported, in 1999, that an increasing BMI was strongly associated with a diagnosis of APL among patients affected by AML; in a cohort of 1245 patients with AML, which included 120 APL, the mean BMI was 27.6 and 25 in APL and no-APL patients, respectively.^31^ At our Hematology Department, in the “Sapienza” University of Rome, 90 patients (62.5%), of a group of 144 consecutive patients, both adults and children, who received the GIMEMA AIDA protocols, were overweight, and of them 66% were over 40 years.

Increased BMI is present in children with APL, but seems to increase with age in all APL patients.^32^

Compared to the disease in adults, childhood APL is more frequently associated with hyperleukocytosis and a higher number of circulating blasts; in spite of this, the outcome results are comparable. It should be noted that the three largest pediatric studies reported a relatively high proportion of children with hyperleukocytosis at presentation, ranging from 35% to 48%. In both GIMEMA-AIEOP AIDA and European APL 93–2000 trials approximately 35% of pediatric patients were classified as high risk patients according to WBC count.^6^,^17^ A significantly higher median value of WBC counts at diagnosis had been previously reported in children by Guglielmi et al. (3.6 ×10^9^/L vs 2,6 ×10^9^/L, respectively in children and adults).^30^ One large recent European APL study, reporting the analysis of 84 children treated with 2 consecutive trials, confirmed this observation and described the difference between children ≤12 vs ≥13 years of age (WBC 10.8 vs 2.6 × 10^9^/l respectively). Using the commonly adopted cut-off value of 10×10^9^/L, the incidence of hyperleukocytosis is clearly higher in children than in adolescents and in adults, in whom it is usually around 20% to 25%.^33^ The 749 adults, who entered the same European APL protocols, had a similar median WBC levels (2.3×10^9^/l) as adolescents; thus the biggest difference in WBC count at onset is observed between young children and patients aged more than 13.^28^,^33^ Also in smaller series of children with APL, a WBC count at presentation over 10 ×10^9^/l seems to be more common; in the first French childhood series, 13/31 (42%) children had WBC greater than 10.0 × 10^9^/l, and 7 of them presented WBC over 25.0 × 10^9^/l at diagnosis.^8^ In the BFM pediatric APL series, 30/81 children (30%) presented WBC ≥ 10×10^9^/l, with 3 of them showing leucocytes count ≥ 100×10^9^/l.^14^ In the Japanese series of 58 children with APL, 35 (60%) had hyperleukocytosis at disease presentation.^34^

Other characteristics, such as the microgranular M3 variant (M3v according to French-American-British – FAB-classification) and the promyelocytic leukemia/retinoic acid receptor-alpha (PML/RARα) isoforms bcr2 and bcr3 have been reported with increased incidence in children (25% and 37.5%, respectively) as compared to adults (12% and 25%, respectively), although isoform bcr1 remains the most common in all age groups.^8^,^28^ A higher prevalence of M3v morphology (32% vs 16.5%) and the bcr3 type of PML/RARα transcript (56.5% vs 35.5%) have been reported for pediatric APL in some series.^30^ However the association of M3v and bcr2 and bcr3 isoform with pediatric age is not clearly defined and not always reported in very recent trials. Bally et al. found higher incidence of M3v in patients with APL aged < 18 ( 23% ≤12 y, 24% 13 to 18 y; 13 % 19 to 60 y; p=0.03) but no difference regarding PML/RARα isoforms (bcr1: 50% ≤12 y, 62% 13 to 18 y; 61 % 19 to 60 y; bcr2: 14% ≤12 y, 9% 13 to 18 y; 9 % 19 to 60 y; bcr3: 36 ≤12 y, 29% 13 to 18 y; 30% 19 to 60 y; p= 0.79).^33^ Similarly, M3v and the PML/RARα isoforms bcr2 and bcr3 were found not to be increased in the AIEOP-GIMEMA and PETHEMA studies when compared to adults. In fact, the incidence of 18% and 43% of M3v and of the bcr3 isoform in the PETHEMA pediatric series do not differ from the 19% and 44% reported for the whole series of APL patients included in the PETHEMA LPA96 and LPA99 studies.^2^,^3^,^5^–^7^

A low incidence of additional cytogenetic rearrangements has been reported in pediatric APL by Raimondi at al. and in children included in the European APL93 trial (11% of children vs 27% of adults carrying chromosomal abnormalities in addition to PML/RARα).^35^ On the contrary, these findings were not confirmed by Ortega at al. in the PETHEMA LPA96 and LPA99 trials in which the proportion of children with additional chromosomal abnormalities did not differ from that reported in the PETHEMA study of adult patients.^2^,^3^,^7^

FMS-like tyrosine kinase (FLT3) mutations have been examined as a prognostic indicator in adult and pediatric APL. Mouse models have demonstrated that FLT3 mutations cooperate with RARα translocations by conferring a proliferative advantage to cells in maturation arrest. The differentiation arrest caused by t(15;17) likely cooperates with the proliferative advantage conferred by FLT3 mutations in APL development and/or progression. Studies of APL patients (mostly adults) have shown that 20–30% of patients harbor in their leukemic cell the FLT3/internal tandem duplication (ITD) and another 10–20% carry the FLT3/tyrosine kinase domain (TKD) mutation. As to the prognostic significance of FLT3 mutations in APL, there is no consensus at present and divergent conclusions have been reported in the published studies. The European cooperative APL Group found that there was a trend toward shorter overall survival in patients with FLT3/ITD (but not in those with FLT3/TKD) due to very poor post-relapse survival.^36^ On the contrary, no correlation between FLT3 mutations and survival have been found by Stock, et al. in 78 adult patients treated on Cancer and Leukemia Group B (CALGB) C9710.^37^ Nevertheless in the UK Medical Research Council (MRC) AML10 and AML12 trials that included 203 adult and children with APL, patients with FLT3 mutations [both FLT3/ITD and missense mutations in the activation loop domain of the tyrosine kinase domain (FLT3/ALM)] had a higher rate of induction death but no difference in relapse risk or overall survival.^38^ The prevalence and the prognostic significance of FLT3 mutations have not been well defined in childhood APL. One earlier study examined FLT3 mutations in a pediatric APL population; among 29 children, FLT3 mutations were present in 10 (34.5%) of them and were strongly associated with higher leukocyte count.^39^ The largest study on FLT3 mutations restricted to pediatric patients with APL examined 104 patients aged < 21 years; 81 treated within cooperative group trials CCG-2891 (n=13), CCG-2911 (n=18) and CALGB C9710 (n=50) and 23 treated according to institutional standard therapy.^40^ This study demonstrated a high prevalence of both FLT3/ITD and FLT3/TKD mutations in childhood APL (40%). Furthermore, a strong correlation between FLT3 mutations and WBC count at diagnosis (median diagnostic WBC count for children with FLT3 mutations 32.95 × 10^9^/l compared to 3.6 × 10^9^/l in those with wild-type FLT3 -p=0.004) and a significantly higher proportion of M3v in FLT3 mutated compared to FLT3 wild type patients (47% versus 15%, p=0.035) were found. In the same study, analysis of induction death by FLT3 mutational status in the high WBC count group showed an early death rate (EDR) of 47% and 0% in FLT3 mutant and FLT3 wild type patients, respectively (p=0.052). The association of FLT3 mutant genotype with induction death in patients with higher WBC count may indicate direct contribution of the FLT3 activation to coagulation dysregulation. If the link between FLT3 mutated status, coagulopathy and induction death observed in this study is further substantiated, interruption of the FLT3 signal transduction pathway by FLT3 inhibitors may represent an attractive therapeutic strategy to ameliorate the rapidly progressive coagulopathy and counteract early death risk.

Other reported characteristics of childhood APL, compared to adults, include more frequent organomegaly and a higher incidence of the expression of the T-antigen CD2 and of the stem cell marker CD34, which are generally also correlated with bcr3 isoform and M3v.^30^

The true incidence of central nervous system involvement (CNS) at diagnosis is unknown both in children and adults. One small pediatric study described initial CNS leukemia in 3/40 (7,5%) patients while a large more recent pediatric trial reported an incidence of less than 2–5%.^6^–^8^;^14^,^41^ Patients with APL commonly present with coagulopathy. Approximately 80% of them have a prolonged INR, elevated fibrinogen degradation products, low fibrinogen, and thrombocytopenia, all potential causes of the severe hemorrhages. Therefore, it is recommended in modern guidelines that lumbar puncture at diagnosis is not performed in light of the high risk of bleeding.

## Current Treatment Approach in Childhood APL

Specific therapeutic strategies for pediatric APL have been derived from adult trials that included children. Most of these approaches include the simultaneous combination of ATRA and anthracycline-containing chemotherapy. In the firstly published study of a German-Austrian-Swiss group, 95% of the 22 children treated with ATRA followed by chemotherapy achieved CR and the 5-year Overall Survival (OS) and Event-Free-Survival (EFS) were 87% and 76% respectively.^29^ The European APL93 study included 31 children receiving ATRA followed by or combined to daunorubicin and cytarabine; the CR rate was 97%, and the 5-year OS and EFS were 90% and 71%, respectively.^8^ The Italian GIMEMA-AIEOP AIDA-0493 trial (ATRA and idarubicin as induction followed by 3 polychemotherapy consolidation courses), the largest pediatric APL series during the ATRA era, reported a CR rate of 96% and a 10-year OS and EFS of 89% and 76% respectively. Similar results were obtained in children treated with PETHEMA LPA96 study that included the same idarubicin and ATRA combination followed by three anthracycline-based consolidation courses (CR 92%; OS 71%).^7^ The main characteristics and therapeutic results of these studies are summarized in Table 1. All of these studies confirm the virtual absence of leukemia resistance using state-of-the-art treatment. Sample size, eligibility criteria, and some differences in patients characteristics with potential impact on responses to therapy can explain the apparently different results, which are not statistically significant.

Since the introduction of ATRA, the EDR, mainly because of bleeding, is dramatically reduced in the pediatric APL studies (3–7%); similar results have been reported in most of adult cooperative group trials (5–10%).^5^,^9^ However significant higher EDR have been recently observed in unselected population-based studies by Park^42^ et al. and Lehamann et al.^43^ (17.3% and 29%, respectively, with a higher incidence for older patients), in which all adult patients with newly diagnosed APL are reported. The apparently lower EDR in adults and especially in children enrolled in the clinical trials may partially reflect earlier referral to specialized centers, without delay in ATRA administration. In children, the fewer age-related comorbidities could explain the lower EDR. However, to better establish the size of the problem, all authors of clinical trials studies should be requested to report all information for patients who were excluded from the study because of eligibility criteria.

The APL trials conducted in the 1990–2000 decade provided an important source for the investigation of prognostic factors to be used for treatment stratification. In particular the so called Sanz’s score for the relapse risk (low: initial WBC < 10×10^9^/l and platelet count > 40×10^9^/l; intermediate: WBC < 10×10^9^/l and platelet count ≤ 40×10^9^/l; high-risk WBC ≥10×10^9^/l) was developed to dissect relapse risk categories for patients receiving AIDA-like regimens adopted by the GIMEMA and PETHEMA groups^1^,^9^. This in turn allowed the design of distinct strategies which were aimed at sparing unnecessary toxicity for patients with low-risk (WBC < 10×10^9^/l), whereas more intensive post induction chemotherapy including cytarabine were adopted for high risk (WBC > 10 ×10^9^/l) patients. The results of both GIMEMA and PETHEMA trials using a risk adapted approach for adult APL, showed a significant improvement in patient outcome.^18^,^44^ Other large trials conducted by the French European APL group, the British MRC, the Japanese Adult Leukemia Study Group (JALSG) and the German AML Cooperative Group (AMLCG) confirmed the advantage of risk adapted strategies using mainly WBC count as a prognostic factor. Overall, studies reported in recent years with ATRA-based and risk adapted chemotherapy, resulted in CR rates of up to 95% and OS rates > 85% for adult APL.^45^ In the pediatric setting, initial WBC count is the most important prognostic factor influencing the outcome and children with WBC count higher than 10×10^9^/l (who usually have a younger age as compared to children with low WBC counts) have a higher risk of relapse. In the pediatric series of GIMEMA-AIEOP AIDA 0493 trial, a leukocyte count at diagnosis ≥ 10 ×10^9^/l had a negative impact on EFS (59% vs 83% at ten years); the 5-year cumulative incidence of relapse (CIR), among the 61 children treated with PETHEMA trials, was higher for those with presenting WBC ≥10×10^9^/l, compared to those with lower WBC count (31% vs 3.5%).^6^,^7^ The PETHEMA LPA 99 and GIMEMA AIDA-2000, risk-adapted trials, were adopted also in the pediatric population and confirmed the improvement in results as reported in adults. For children in the previous PETHEMA LAP96 study, the 5-year disease-free survival (DFS) was 75%, whereas, in the LPA99 study, it was 89%.^7^ The 6-year OS and DFS rates for the 123 children treated with AIDA 2000 risk-adapted regimen resulted superior compared to those achieved in children who received the AIDA 0493 protocol (96% vs 89.7% and 82.5% vs 73.1%, respectively). For the low-risk children, the less intensive anthracycline-based plus ATRA consolidation was equally effective as the previous cytarabine-containing regimen (6-year OS and DFS 94.2% and 95.6% vs 76.7% and 82.7%, respectively). The role of ATRA combined with cytarabine and anthracyclines during consolidation resulted in a significant improvement in OS and DFS in the high-risk group (96.8% and 82.3% vs 81.6 and 65.2, respectively for AIDA-2000 vs-0493).^17^

As mentioned above, the incidence of CNS involvement in APL, both at diagnosis and relapse, remains to be established, as does the need for prophylactic intrathecal chemotherapy in children and adolescents as an integral part of first-line therapy. Approximately 10% of relapses have a CNS component.^1^ The risk of CNS involvement seems to be extremely low in patients without hyperleukocytosis at diagnosis and, in any case, it is more frequent for those with initial WBC > 10 ×10^9^/l. Other risk factors for CNS recurrence remain a controversial matter. Some authors have previously suggested that FLT3-ITD mutation, which correlate with hyperleucocytosis and an increased expression of adhesion molecules, such as CD56, can promote leukemic infiltration in the CNS. In the PETHEMA LAP96 and LAP99 studies, CNS relapse was associated with CNS hemorrhage before or during induction treatment, which emerged as a novel and independent prognostic factor.^46^ This has not been reported before and could have potential therapeutic implications. Therefore, CNS prophylaxis could be considered at least for high-risk patients. In addition, high-dose cytarabine, that ready penetrates the blood-brain barrier could represent a valuable tool to prevent CNS involvement in APL.

## Has Cytarabine, at High Doses, a Role in the Consolidation Treatment of Children with APL?

The PETHEMA-LPA2005 study suggested that the addition of high-dose cytarabine (1 g/m^2^/day × 4 days) to consolidation therapy could reduce the incidence of relapse in patients defined at high risk. Other European adult trials have confirmed this finding.^44^,^45^,^47^ Two questions remain unanswered: 1) can cytarabine replace anthracycline in the consolidation treatment of APL? 2) is there any benefit of adding cytarabine to the consolidation schedules? Both questions are a matter of investigation in children, mainly in those at high-risk. The 5-year DFS of PETHEMA LPA96 (anthracycline monotherapy in consolidation) and GIMEMA-AIEOP AIDA 0394 (polychemotherapy combination with high-dose cytarabine in consolidation) showed no clear difference in the outcome of pediatric APL (5-year EFS 76% vs 77%).^6^,^7^ Luo et al. suggested that the children included in the PETHEMA trials (without cytarabine) had a significant higher EFS (3.5-year EFS 79.6% vs 37.5%; p 0.012), lower frequency of sepsis during treatment (7.7% vs 78.8%; p 0.0015) and lower hospitalization cost than those treated with protocols containing high-dose cytarabine (USA $ 4,700 vs 20,000; p < 0.0001).^48^ On the contrary the BFM protocol, in line with other reports, combined cytarabine at intermediate-high dose to anthracyclines for consolidation therapy of pediatric APL and reported 5-year EFS and OS rates of 73% and 89%, respectively.^14^ These results support the efficacy of high-dose cytarabine in combination therapy for pediatric APL. In the Japanese childhood acute myeloid leukemia cooperative study (AML99-M3), cytarabine was combined with ATRA and anthracycline both in induction and consolidation; the 7-year EFS and OS of the 58 enrolled children were 91% and 93%, respectively, and the CIR plateaued at 3.6% after 2 years. In this last trial, the addition of ATRA in the consolidation phase also contributed to improve the results (Table 2).^34^ Furthermore, the European Leukemia Network recommended including at least one consolidation cycle of high-dose cytarabine for young high-risk patients.^1^

## Are there Particular Treatment Issues for Children with APL?

The optimal pediatric dose of ATRA has not yet been established; the idiopathic intracranial hypertension, commonly called pseudotumor cerebri (PTC), can complicate the treatment of APL with ATRA. The diagnosis of PTC is based on increased intracranial pressure with normal cerebrospinal fluid composition and negative cerebral imaging studies (computed tomography or magnetic resonance imaging scan). This side effect is more common in children and adolescents, but the incidence decreases with the use of a lower dose of ATRA, without apparently compromising the outcome results. In the European APL 93 trial with the dose of 45 mg/m^2^ severe headache episodes were more frequent in the pediatric population than in adults (16% vs 1–2%).^8^,^28^ Several studies have also reported increased neurotoxicity of ATRA in children, particularly in younger age (<10 years). In an attempt to reduce ATRA related toxicity the daily dose administered in children treated according to GIMEMA-AIEOP AIDA and PETHEMA protocols, was reduced to 25 mg/m^2^.^6^,^7^ This dose proved to be equally effective with a lower incidence of side effects in a previous adult APL dose reduction trial. Available data also suggest that a half dose of ATRA can be as effective as the standard dose of 45 mg/m^2^ per day.^49^ The apparently lower incidence of PTC and headache, together with the excellent therapeutic results obtained with ATRA at 25 mg/m^2^ suggest that such dose could be the recommended standard for children.^50^

Another issue of particular importance in children with APL is the difficulty in swallowing the 10-mg gel caps of ATRA, since the medication is not available in liquid form. Some studies suggest that the contents of an ATRA capsule can be mixed with milk and administered in a nasogastric tube, for comatose patients, reaching a high serum level.^50^,^51^ Similarly, in very young children it is possible to soften capsules in warm milk and so chew them alone or mixed in a spoonful of soft food. An intravenous liposomal formulation of ATRA has been tested in newly diagnosed patients with APL unable to swallow or absorb medications and has been shown to be effective in inducing CR. Unlike oral ATRA, liposomal ATRA was able to produce molecular CR without addition of chemotherapy. In patients with newly diagnosed APL, using polymerase chain reaction (PCR) assay with a sensitivity level of 10^−4^, Estey et al reported that liposomal ATRA monotherapy induced molecular CR in a significant fraction of patients within 3 months. In more than 30% of patients, PCR negativity persisted for years thereby indicating that liposomal ATRA as a single agent may be curative in APL. However, this ATRA formulation is no longer available.^52^

A relevant problem, linked to the use of chemotherapy in the pediatric population, is a risk of cardiomyopathy, a real threat for children with APL treated with regimens that include high doses of anthracyclines. As suggested by Van Dalen et al, the risk of developing clinical heart failure is dose-dependent, increasing from 0% for 150 mg/m^2^ of cumulative anthracycline dose, up to 14.3% for doses of 600 mg/m^2^.^53^ Relatively high-dose anthracyclines (450–750 mg/m^2^) used in modern chemotherapy plus ATRA regimens have proven successful to achieve high cure rates in adults and children with APL, although the high cumulative anthracycline dose was potentially associated with high risk of late cardiotoxicity.^4^,^6^,^7^,^44^ Although no severe acute cardiotoxicity was observed in our first GIMEMA-AIEOP study, longer follow-up is needed to define the late cardiotoxicity of anthracycline regimens. Late subclinical cardiotoxicity was observed in 52% of the adult survivors of APL treated on the GIMEMA AIDA-0493 and-2000 protocols.^54^ To reduce the risk of developing clinically significant cardiotoxicity and heart failure, which is approximately 5% at 15 years after anthracycline therapy for childhood cancer, the AML BFM study group limited the cumulative anthracycline dose to 350 mg/m^2^ in most APL patients obtaining results comparable to those reported for studies with higher doses.^14^ In the PETHEMA 2005 study, a lower anthracycline dose provided equivalent efficacy with less myelosuppression in patients at low and intermediate risk.^44^ Based on these findings, the International Consortium for Childhood Acute Promyelocytic leukemia (ICC APL) was established and a new study (ICC APL 01) was launched and is still actually ongoing with the goal to investigate the safety and efficacy of a regimen with a reduced cumulative anthracycline dose of 355–405 mg/m^2^ in combination with ATRA and a high dose of cytarabine in one consolidation course for low risk and two courses for high risk patients. Due to the obvious concerns of irreversible heart failure in children who receive high cumulative doses of daunorubicin, also the CALGB C9710 study utilized a cumulative dose of daunorubicin of 500 mg/m^2^ for those >15 years of age and 400 mg/m^2^ in children 3–14 years of age.

## Role of Arsenic Trioxide (ATO) in Pediatric APL

Treatment strategies for childhood APL aim to decrease the incidence of relapse and chemotherapeutic toxicity. The introduction of ATRA has been crucial for both antileukemic efficacy in APL and for reducing EDR. Furthermore, in more recent studies, Arsenic Trioxide (ATO), first introduced for the treatment of relapsed patients has resulted highly effective in achieving high cure rates in association with reduced toxicity in adults with APL.^55^–^59^

Arsenic compounds had been used as therapeutic agents for more than 2000 years in Western and Eastern medicine, particularly in China. Despite the advent of new cytotoxic drugs the empirical use of arsenic as an antileukemic agent continued in China throughout the past century leading ATO to be introduced into the treatment of APL in the 1970s. The specific mechanism of ATO in APL treatment is still under investigation. A dual mechanism of action has been described; as demonstrated by Chen et al^55^ and Shao et al,^56^ at low concentration ATO exerts a partial differentiating effect by inducing the stimulation of both PML-RARα and PML leading to the degradation through the proteasome pathway. On the contrary at high concentrations, ATO induces apoptosis through caspase activation, reactive oxygen species (ROS) production and induction of mitochondria mediated intrinsic apoptotic pathway. Several studies have shown the benefits of ATO in the treatment of relapsed APL, with a remission rate of 80–90% and long term DFS of 60–80%, and also as induction and consolidation therapy for adults with newly diagnosed APL, demonstrating that ATO is the most active single agent in this disease.^55^–^59^

However, experience with ATO for treating pediatric APL is limited compared to that achieved with adults APL studies. Two small but significant series^27^,^60^ reported the use of ATO a single agent for both remission induction and post-remission therapy in children with newly diagnosed APL. In both studies, the morphological CR rate was approximately 90% (91% and 89.5% respectively in George et al^60^ and Zhou et al^27^), and no resistant cases were recorded. The use of ATO as a single agent in post-remission therapy led to an estimated 5-year OS and EFS of 91% and 81% and 84% and 73%, respectively in George’s^60^ and Zhou’s^27^ series, respectively. These results were comparable with those achieved with ATRA plus chemotherapy. ATO-related toxicity was minimal and transient during induction, and neutropenia was the most common side-effect during the 3-year post-remission ATO therapy.^27^,^60^

A striking convergence regarding the antileukemic effects of ATRA and ATO is the degradation of PML/RARα through distinct pathways, with ATRA targeting the RARα and ATO targeting the PML moieties of the fusion protein. The combined use of ATRA and ATO synergistically induces differentiation, apoptosis and accelerates tumor regression in vivo. Shen et al^61^ suggested that ATRA-ATO combination for treating adult APL significantly shortened the time to achieve CR, reduce the disease burden and improved DFS compared with approaches based on the use of either ATRA or ATO alone^61^. These benefits of combining ATRA and ATO have been confirmed by several groups in adults and, more recently, in pediatric APL.^27^,^62^ The retrospective analysis at Pediatric Department, Pecking University, Medical School, showed that the application of ATRA and ATO as induction and consolidation therapy for newly diagnosed children with APL resulted in excellent outcomes and improved the long-term prognosis. Children treated with ATRA-ATO combination had significant better CR and EFS rates, compared to those who received ATRA-based regimes (CR 95.3% and 80%; EFS 92.5% and 70.4%, respectively). Arsenic was well tolerated in children and was devoid of major acute side-effects.^63^ Table 3 summarizes the results in pediatric APL with ATO as first-line therapy.

The long-term safety of ATO in children is unclear. Reported late effects include hyperpigmentation, neutropenia, muscular atrophy and peripheral neuropathy. One study reported a significant association between the children’s neurocognitive function and their chronic environmental ATO exposure.^50^

## What is the Role of Stem Cell Transplant in Pediatric APL?

Several large multicenter studies have shown that regimes combining upfront ATRA and chemotherapy and/or ATO lead to a high curability rate in APL, suggesting that hematopoietic stem cell transplantation (HSCT) is not recommended as consolidation therapy for patients in first complete remission (CR1). However, despite optimal therapeutic results, relapses still occur in 15% to 25% of the cases.

While, for patients relapsing after ATRA plus chemotherapy, ATO as salvage for re-induction is an established recommendation, the post-consolidation approach to relapsed or refractory APL is less clearly defined. Although most relapsed patients (> 80%) can achieve a second CR (CR2) with ATRA, ATO, chemotherapy (CT), or a combination of these agents, only occasional cases have demonstrated second remission for as long as 8–10 years following treatment with chemotherapy and/or ATRA.

For this reason most relapsed APL patients would receive autologous (auto), allogeneic (allo) or haploidentical cell transplantation (HSCT). Auto-HSCT is one of the therapeutic options for patients who achieve a CR2 and are in molecular remission (MR). Allo-HSCT may be useful to consolidate patients in CR2 and to treat patients with persistent minimal residual disease due to a potent graft versus leukemia effect, the greatest asset of an allo-HSCT in this setting.^64^

In the largest study by Sanz et al,^65^ the investigators retrospectively analyzed outcomes in 625 patients with APL who had been treated as part of the European Cooperative Group for Blood and Marrow Transplantation (EBMT) and who underwent either an auto-or allo-HSCT. In this registry survey, similar outcomes were shown for both auto and allo-HSCT, although the allo-HSCT showed a higher incidence of non-relapse mortality (transplant related mortality TRM), whereas the auto-HSCT group had a higher risk of relapse. This study including patients in CR1 or CR2 suggested that, even in the ATRA era, HSCT had to be considered as consolidation therapy, especially for patients in CR2.^65^

The majority of reports on the use of HSTC for treatment of relapsed or refractory APL deal primarily with adult patients making the benefit of these therapies for children still unclear. The Dana Farber Cancer Institute reported a favorable outcome among children undergoing allo-HSCT for relapsed and refractory APL with a probability of survival at 5 years of 73%. This group also reported a TRM of 33% and a low relapse rate (15%).^66^ The largest study by Dvorak et al.^67^ reported 32 pediatric patients underwent auto or allo-HSCT for treatment of primary-refractory (3 patients) or relapsed (29 patients) APL. The incidence of TRM and relapse for auto-transplant in children were 0% and 27%, and the 5 year EFS and OS of 73% and 82%, respectively; for the allo-HSCT the incidence of TRM and relapse were 19% and 10%; the 5 year EFS and OS were 71% and 76%, respectively. This study demonstrates that auto or allo-HSCT are both effective therapies for the treatment of children with relapsed or refractory APL without significant difference in EFS and OS but, as previously described in adults, with a low TRM for auto-HSCT and a low incidence of relapse for allo-HSCT.^67^ Notwithstanding the successful results, the limits of the above mentioned studies are the retrospective nature, the relatively small number of patients included, mainly as concerns pediatric age, and the different therapeutic strategies, according to the single institute policy.

The optimal stem cell transplantation strategy for advanced APL still remains controversial, as several factors influence the choice. The type of salvage regimens, donor selection, conditioning regimens and graft versus host disease (GVHD) prophylaxis should be included in the context of prospective clinical trials. However, the superior protection from relapse afforded by allo-HSCT and the favorable survival described in the few pediatric experiences reported, suggest performing allo-HSCT for children with relapsed or refractory APL for whom a suitable HLA-donor is available. On the other hand, children, without a suitable donor, achieving molecular remissions after salvage therapy can be considered candidates for high dose chemotherapy and auto-HSCT as a valid post-consolidation strategy.

## Future Directions

For patients with newly diagnosed, non-high risk APL, the front-line use of ATRA-ATO combination is extremely encouraging and will probably become the standard regimen also in the pediatric age. Future controlled prospective trials should address the role of ATO in induction and consolidation chemotherapy and establish whether ATO could reduce cytotoxic chemotherapy intensity in children with APL. Oral arsenic is also very effective, and its combination with oral ATRA warrants further investigation also in the pediatric setting.^68^,^69^ Next ATO-based pediatric APL trials should include longitudinal assessment of neurological and neurocognitive outcomes, evaluation of the growth assessment in treated children and pharmacokinetic studies will be necessary to investigate how the faster metabolism in children can promote drug excretion and influence the right dose to administer, in particular in younger patients.

New drugs, such as FLT3 inhibitors, anti-CD33 monoclonal antibodies and tamibarotene (a synthetic retinoid more potent and less toxic than ATRA) may offer additional options for patients with high-risk or relapsed/refractory APL.

Future efforts should focus on decreasing the delay in referral and diagnosis; unfortunately intracranial bleeding is still the major cause of early death and a small percentage of patients, adults and children, in all countries, are still diagnosed after the occurrence of life-threatening bleeding. More importantly, as reported by Park et al,^42^ there is a clear need to provide the knowledge necessary to recognize APL as a medical emergency, which requires specific and simultaneous actions, including a prompt initiation of ATRA, aggressive supportive care to counteract the coagulopathy, and patient referral to experienced medical centers when the disease is first suspected. Finally, more studies are warranted to clarify the reasons for the different epidemiology of pediatric APL in several countries.

## Figures and Tables

**Table 1:** FIRST pediatric protocols (atra+chemo) for pediatric APL.

Year	2001	2004	2005	2006
**Group**	G-A-S	French	AIEOP-GIMEMA	PETHEMA
**No. of Pts**	81	31	124	66
**Induction Therapy**	ATRA+ADE/AIE	ATRA+AD	ATRA+IDA	ATRA+IDA
**ATRA dosage**	25 mg/m^2^	45 mg/m^2^	25 mg/m^2^	25 mg/m^2^
**CR (%)**	95	97	96	92
**ID (%)**	5	3	4	7
**5-year EFS (%)**	76	71	76	77
**5-year OS (%)**	87	90	89	87
**Cumulative anthracycline dose**	DNR 60, IDA 24, ADR120	DNR 495	IDA 80, MTZ 50	IDA 80, MTZ 50/100

**Table 2:** Pediatric protocols for pediatric APL (atra+chemo+hd-ca).

Year	2010	2010
Group	BFM	Japanese Childhood AML Cooperative Study
Induction Therapy	ATRA+IDA+VP/CA+VP+DNR	ATRA+IDA+CA
No. of Pts	81	58
Consolidation Therapy	CA+IDA+HD-CA+VP16	HD-CA+MTZ+ATRA+PIRARUBI CIN+ACLARUBICIN
EFS (%)	73 (5 years)	91 (7 years)
OS (%)	89 (5 years)	93 (7 years)

**Table 3:** Chinese experiences in pediatric APL: ATO as first-line therapy

Author	Year	N. pts	Age yrs	Induction	CR (%)	Post-induct.	Outcome
Zhang	1999–2012	65	13 (med.)	ATRA±ATO	90.8	CHT	5-y EFS 77.5%
							5-y OS 88.9%
Zhou	2001–11	19	4–15 (range)	ATO	89.5	ATO	5-y EFS 72.7%
							5-y OS 83.9%%
Wang	2000–11	35	NA	ATO±ATRA	85.7	CHT	5-y EFS 78.3%
							5-y OS 82.7%
Zhang	2003–12	37	2–14 (range)	ATRA±ATO	94.6	CHT	5-y EFS 79.2%
							5-y OS 91.5%
